# Supine versus Prone 3D Abus Accuracy in Breast Tumor Size Evaluation

**DOI:** 10.3390/tomography8040167

**Published:** 2022-08-12

**Authors:** Anna D’Angelo, Gianluca Gatta, Graziella Di Grezia, Sara Mercogliano, Francesca Ferrara, Charlotte Marguerite Lucille Trombadori, Antonio Franco, Alessandro Cina, Paolo Belli, Riccardo Manfredi

**Affiliations:** 1 Dipartimento di Diagnostica per Immagini, Radioterapia Oncologica ed Ematologia, Fondazione Policlinico Universitario “A. Gemelli”, IRCCS, 00168 Rome, Italy; 2 Dipartimento di Medicina di Precisione, Università degli Studi della Campania “L. Vanvitelli”, 80138 Naples, Italy; 3 Dipartimento di Radiologia, Ospedale “G. Criscuoli”, 83054 San’Angelo dei Lombardi, Italy; 4 Casa di Cura Villa dei Fiori, 80011 Acerra, Italy; 5 Unità di Senologia, Dipartimento della Donna, del Bambino e di Salute Pubblica, Fondazione Policlinico Universitario “A. Gemelli”, IRCCS, 00168 Rome, Italy

**Keywords:** 3D automated breast ultrasound (ABUS), breast cancer, hand-held ultrasound (HHUS), magnetic resonance imaging (MRI), breast imaging

## Abstract

Breast-conserving surgery (BCS) with negative resection margins decreases the locoregional recurrence rate. Breast cancer size is one of the main determinants of Tumor-Node-Metastasis (TNM) staging. Our study aimed to investigate the accuracy of supine 3D automated breast ultrasound (3D ABUS) compared to prone 3D ABUS in the evaluation of tumor size in breast cancer patient candidates for BCS. In this prospective two-center study (Groups 1 and 2), we enrolled patients with percutaneous biopsy-proven early-stage breast cancer, in the period between June 2019 and May 2020. Patients underwent hand-held ultrasound (HHUS), contrast-enhanced magnetic resonance imaging (CE-MRI) and 3D ABUS—supine 3D ABUS in Group 1 and prone 3D ABUS in Group 2. Histopathological examination (HE) was considered the reference standard. Bland–Altman analysis and plots were used. Eighty-eight patients were enrolled. Compared to prone, supine 3D ABUS showed better agreement with HE, with a slight tendency toward underestimation (mean difference of −2 mm). Supine 3D ABUS appears to be a useful tool and more accurate than HHUS in the staging of breast cancer.

## 1. Introduction

Automated breast ultrasound (ABUS) is a standardized technique that provides reproducible, high-resolution images, and operator independence using an automated scanner with a large field of view (FOV). Compared to hand-held ultrasound (HHUS), the main advantage of ABUS is that it achieves multiplanar reconstruction of the breast to improve lesion differentiation by radiologists [1]. ABUS has been increasingly used in clinical practice in recent years as it can overcome some limitations of HHUS, such as operator dependence, and can increase the reproducibility of the examination [2]. The time of image acquisition is different from the moment of image interpretation, reducing operator dependence and time of examination [3]. ABUS was approved by the Food and Drug Administration (FDA) in 2012 as a supplemental screening tool for women with heterogeneous and extremely dense breasts [4]. To date, this is its prevalent use in screening programs [5,6]. Although the evidence for long-term benefits of using ABUS in screening settings is limited, several studies have shown that cancer detection rates have increased from 1.9 to 7.7 cases per 1000 women [7]. The new-generation automated scanners are usually classified into two main categories: prone and supine scanners [8,9]. At present, there are different fields of application in ABUS use, such as second look examination after contrast-enhanced magnetic resonance imaging (CE-MRI); re-evaluation after neoadjuvant chemotherapy; and the estimation of the lesion size, with consequences on the treatment plan, and the curative effect and prognosis. According to ESMO guidelines (2019), BCS is the preferred local treatment option for the majority of early breast cancer patients (tumor ≤ 2 cm), with the exception of aggressive phenotypes and/or positive axilla [10]. Negative resection margins decrease the locoregional recurrence rate [11,12,13]. Breast cancer size is one of the main determinants of Tumor-Node-Metastasis (TNM) staging [14], according to the eighth edition of the *AJCC TNM Staging System* [15]. Local breast cancer staging is currently performed using digital mammography (DM) and digital breast tomosynthesis (DBT), coupled with ultrasonography (US) of the breast and regional lymph nodes [16]. Preoperative CE-MRI remains a controversial issue: it is not routinely recommended but should be considered in some cases. CE-MRI allows for the definition of the extension of the lesion, searching for additional ipsilateral cancers, and screening for contralateral cancers, but it is associated with a higher mastectomy rate [17,18]. The combined use of the three techniques (DM, DBT and US) achieves the overall accuracy of CE-MRI [19]. Several new techniques are being tested for diagnostic imaging, such as ABUS, shear-wave elastography and contrast-enhanced mammography (CEM). These have the potential to increase diagnostic accuracy, especially in women with dense breasts [20,21,22]. Many studies have assessed the advantages of ABUS in the staging of breast cancer [23] and its accuracy compared to HHUS and CE-MRI in measuring the size of the tumor [24,25]. Still, to our knowledge, a comparison between the different ABUS categories has never been performed. Our study aimed to investigate the agreement of supine 3D ABUS compared to prone in the evaluation of tumor size in patients with breast cancer who were candidates for BCS. Histopathological examination (HE) was considered the reference standard.

## 2. Materials and Methods

This prospective two-center study obtained the approval of the local ethics committee of the two hospitals involved: Fondazione Policlinico Universitario “A. Gemelli” (FPG) IRCCS Rome (IT), called Group 1, and University of Campania “L. Vanvitelli”, Naples (IT), called Group 2.

In the period between June 2019 and May 2020, patients with percutaneous biopsy-proven early-stage breast cancer (cT1-2, cN0, M0) [26] eligible for surgery were consecutively enrolled in each institution.

The eligibility criteria were 18 years or older, and percutaneous biopsy-proven unifocal early-stage breast cancer. The exclusion criteria were previous history of breast surgery (e.g., breast surgery up to 12 months before breast cancer diagnosis), preoperative neoadjuvant chemotherapy, histological examination not available/incomplete and mammography-only visible cancers. Participants were enrolled after written informed consent was obtained.

A final diagnosis was obtained by HE after mastectomy or breast-conservative surgery for all index lesions. HE was considered the reference standard to define cancer size by the maximum diameter measured on fresh surgical specimens.

### 2.1. Imaging Protocols

**Figure 1:**
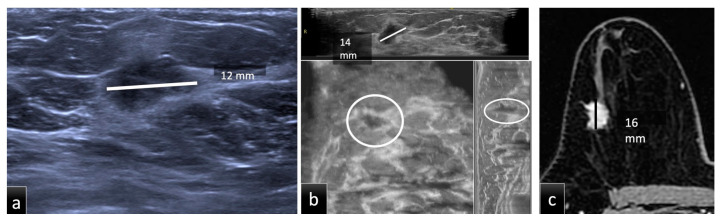
Group 1, a 51-year-old patient with biopsy-proven ductal carcinoma in situ (DCIS) in the upper outer quadrant of the right breast. The maximum diameter provided by HHUS (**a**) was 12 mm, while on CE-MRI, (**c**) it was 16 mm. Supine 3D ABUS (**b**) in the axial plane, coronal reconstruction and sagittal reconstruction showed an inhomogeneous hypoechoic mass with irregular margins and the largest diameter of 14 mm. The histopathological size was 16 mm.

**Figure 2:**
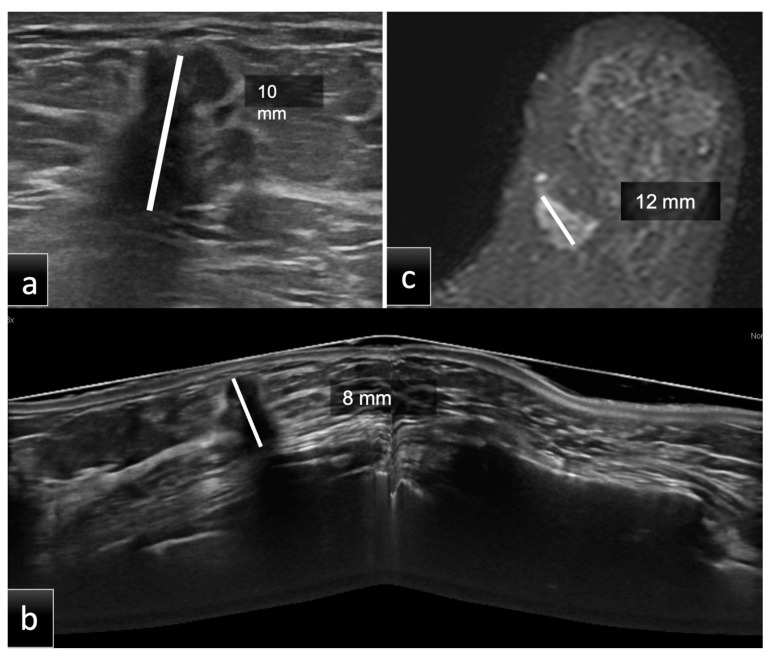
Group 2, a 60-year-old patient with histological diagnosis of invasive ductal carcinoma (IDC) in the lower inner quadrant of the left breast. The maximum diameter measured on HHUS was 10 mm (**a**). At the same time, on CE-MRI, (**c**) the lesion had a maximum diameter of 12 mm. Prone 3D ABUS in the axial plane (**b**) showed a hypoechoic mass with irregular margins and the largest diameter of 8 mm. The histopathological size was 10 mm. ABUS and HHUS examinations were performed in both groups during the same study session by two different dedicated breast radiologists.

Patients underwent breast examinations including HHUS, DM, CE-MRI and supine 3D ABUS in Group 1 and prone 3D ABUS in Group 2 (Figure 1 and Figure 2).

#### 2.1.1. Hand-Held Ultrasound (HHUS)

HHUS acquisitions were performed in Group 1 using an ACUSON S2000 (Siemens Medical Solutions, Inc., Mountain View, CA, USA) with a linear transducer and a bandwidth of 12–18 MHz, adopting a free-hand positioning technique.

In Group 2, HHUS examinations were performed with a dedicated breast US unit, LOGIQ S6 (GE Healthcare, Milwaukee, WI, USA), equipped with an M12L 5–13MHz probe, adopting a free-hand positioning technique.

#### 2.1.2. Supine and Prone 3D ABUS

Supine 3D ABUS scans were performed with an ACUSON S2000; prone 3D ABUS scans were performed with SOFIA™ (iVu Imaging Corporation, Southlake, TX, USA).

-Supine 3D ABUS examinations were acquired in at least three scan views (lateral, anteroposterior and medial), and up to five views (superior and inferior), using a 5–14 MHz wide linear array ultrasound transducer mounted on a flexible arm. An automated one-button pressure and locking mechanism was used to speed up and simplify the acquisition technique. The most suitable setting for each patient was selected according to their breast size (A–D preset cup sizes; cup A = 11 MHz, cup B = 10 MHz, cup C = 9 MHz, cup D = 9 MHz and cup D^+^ = 9 MHz). A replaceable membrane covered the transducer, and a specific lotion was used to avoid air bubble formation at the contact surface with the skin. Once the imaging data were obtained, they were processed using computer algorithms and stored on a hard drive. The axial image series were sent to a dedicated workstation and combined to form a 3D US image that can be examined in multiplanar reconstructions.

-Prone 3D ABUS consisted of a scanning table of 184 mm in diameter, into which the ultrasound transducer was embedded, and a 3D review and documentation workstation. The operator distributed a thin layer of acoustic gel on the examination disc, which had a built-in Hitachi probe. After applying some gel to the breast, the patient got on the examination table and placed the breast in the disc with the nipple positioned in the center. The patient lay prone with the contralateral leg bent in order to rotate the nipple slightly in the cone center; the arm could be placed by the patient’s side. A 92 mm-long L53L transducer automatically revolved around the breast and captured an entire breast in a single revolution (30 s per breast). The pre-scan modality allows for optimization of patient positioning (i.e., smallest drop-off artifact possible or patient repositioning) and modification of the scan parameters (gain, depth, focus, etc.). SOFIA acquisition requires, on average, 10 min per patient. Ultimately, data were sent to the workstation and independently reviewed by two dedicated breast radiologists.

#### 2.1.3. Contrast-Enhanced Magnetic Resonance Imaging (CE-MRI)

In Group 1, CE-MRI was performed using a 1.5-T MRI scanner (Optima MR450w GEM; GE Medical System, Milwaukee, WI, USA), with an 8-channel bilateral breast coil. Patients were scanned in the prone position. MRI imaging was assessed using the protocols described below. An intravenous cannula was inserted into the cubital vein prior to the investigation for contrast administration. Dynamic contrast enhancement imaging was performed with one pre-contrast acquisition, followed by five consecutive scans after intravenous administration of 0.2 mmol/kg of Gadoteridol (ProHance, Bracco, Milan, Italy) using a power injector at a flow rate of 2 mL/s, followed by a 20 mL saline flush. The following sequences were acquired: propeller axial sequence (TE 104, TR 7410, FOV 36 cm, NEX 2, 3 mm thickness, 0, 3 spacing, 512 × 512), DWI axial sequence (TE 63.9, TR 9362, FOV 36 cm, NEX 1, 3 mm thickness, 0, 3 spacing, 256 × 256, B1000), 3D VIBRANT axial sequence (TE 2.2, TR 6.8, FOV 37 cm, FA 10, NEX 0, 1.6 mm thickness, 512 × 512), 3D VIBRANT sagittal sequence (TE 2.2, TR 7.4, FOV 25 cm, FA 12, NEX 0, 2.4 mm thickness, 512 × 512).

In Group 2, CE-MRI was performed with a 1.5-T unit (Symphony, Siemens Healthcare, Erlangen, Germany). All patients were studied in the prone position using a dedicated breast coil. The MRI protocol included a transverse T1 localization sequence, a sagittal T2 sequence with fat saturation and a T1 gradient echo sequence 3D-FLASH (TE 2.5 ms, TR 12.7 ms, acquisition time less than 2 min, FA 30°, slice thickness 3 mm, interslice gap 0 mm, NEX 1, FOV 35, matrix size 256 Å~160, pixel size 1.4 Å~1.4), with a series of six coronal acquisitions. After intravenous administration of Gadobutrol (Gadovist, Bayer Schering Pharma AG, Berlin, Germany) with bolus injection of 0.1 mmol/kg of body weight, images were acquired starting at 0, 2, 4, 6 and 8 min.

### 2.2. Image Analysis

The measurements of breast cancer size were performed in the same way in both groups to reduce bias.

The imaging examinations in Groups 1 and 2 were assessed by breast radiologists with 5 and 10 years of experience, respectively. The radiologists were completely blinded to any clinical information or previous radiological data. There were three reading sessions according to the different examination techniques (HHUS, ABUS and CE-MRI), separated by at least three weeks to avoid memory bias. The measurements were conducted blinded to the methods and histology. As illustrated in Figure 1 and Figure 2, the tumor size of each lesion was measured in millimeters (mm), as the largest lesion diameter found on native or multiplanar supine 3D ABUS, prone 3D ABUS and CE-MRI views, as well as manual HHUS scans. On MRI, tumor size was measured on the second scan acquired after contrast administration. Size analysis was performed on the total number of lesions and then stratified into three groups according to tumor size:

- Group A (size less than 10 mm);
- Group B (size between 10 mm and 20 mm);
- Group C (size more than 20 mm).

### 2.3. Statistical Analysis

The statistical analyses were conducted with the following software programs: “BA-plotteR-A web tool generating Bland-Altman plots and constructing limits of agreement” (https://doi.org/10.1016/j.rvsc.2021.05.017) (accessed on 11 July 2022), and IBM SPSS Statistics for Windows, version 26 (IBM Corp., Armonk, NY, USA).

Statistical evaluation was performed using the Bland–Altman analysis (B&A) and plots. B&A was performed to highlight both similarities and differences between the different measurement methods, revealing if one method overestimates high values or underestimates low values, and graphically representing, through the distribution of the scatter plot, if a repeating type of error, such as a systematic error or random relative error, is present.

The HE measures in mm were considered the gold standard for comparison. Preoperative imaging evaluations performed through supine and prone 3D ABUS in Groups 1 and 2, respectively, were compared to the reference standard to quantify their level of agreement. The measurements collected with HHUS and CE-MRI in the two groups were evaluated together and compared to the reference standard.

## 3. Results

The study cohort included 88 patients, 44 for each arm. One hundred patients diagnosed with breast cancer between June 2019 and May 2020 were considered for the study. Twelve patients were excluded because six received neoadjuvant chemotherapy, and six underwent surgery in different hospitals.

In Group 1 (mean age, 56.2 years; range, 40–77 years; SD 10.9), 40 patients underwent BCS, and 4 patients underwent mastectomy.

In Group 2 (mean age, 60.5 years; range, 33–79 years; SD 10.7), 37 patients underwent BCS, and 7 underwent mastectomy.

The histological type of breast cancer was analyzed. Lesions were divided into subtypes based on histology: invasive ductal carcinoma (IDC); ductal carcinoma in situ (DCIS); invasive lobular carcinoma (ILC); no special type carcinoma (NST).

The molecular subtype evaluating the expression in tumor tissue of hormone receptors (estrogen receptor (ER) and progesterone receptor (PR)), human epidermal growth factor receptor type 2 (HER2) and Ki67 was considered. In addition, the lesion side (right or left breast) and lesion localization (quadrant of the breast) were evaluated. For each examination, radiologists defined the result of examination for each side (left and right breast), and the quadrant or the lesion site. We considered the correspondence between the breast quadrant and clock position to optimize the interobserver concordance, morphology, margins and side (left or right) [27]. Table 1 details the characteristics of the two populations, showing no statistically significant differences between the two groups (*p* > 0.05, Student’s *t*-test), especially regarding patients’ age and the histological tumor size.

### 3.1. Imaging Analysis

#### 3.1.1. Assessment of Tumor Size with HHUS, 3D ABUS and Histology

Table 2 illustrates the mean lesion size measured by different imaging modalities (HHUS, 3D ABUS, CE-MRI) and HE in the two groups.

#### 3.1.2. Analysis of Agreement between Each Modality and Histology

##### Supine 3D ABUS

Table 3 illustrates individual measurements obtained by supine 3D US and HE. The comparison between the 44 preoperative measurements and the HE led to a mean difference (*d*) of −2 mm and upper and lower limits of agreement (LoAs) of 2 and −9.55, respectively, as reported in Table 4.

**Figure 3:**
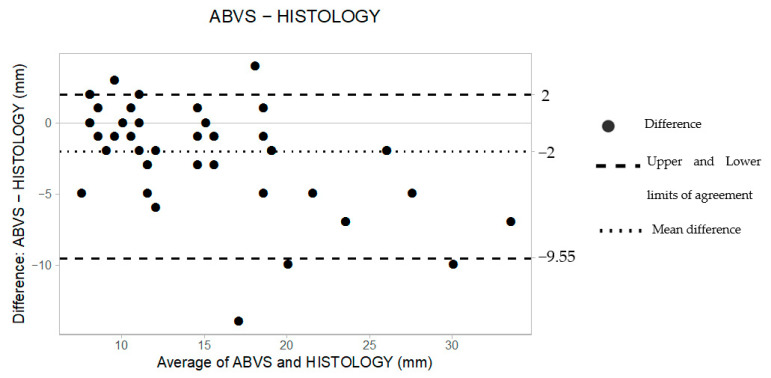
The Bland–Altman scatter plot shows the agreement of measurements between supine 3D ABUS (ABVS) and histology. Millimeters (mm).

The B&A scatter plot also shows that 95% of the data lie between the constructed LoAs (Figure 3).

##### Prone 3D ABUS

**Figure 4:**
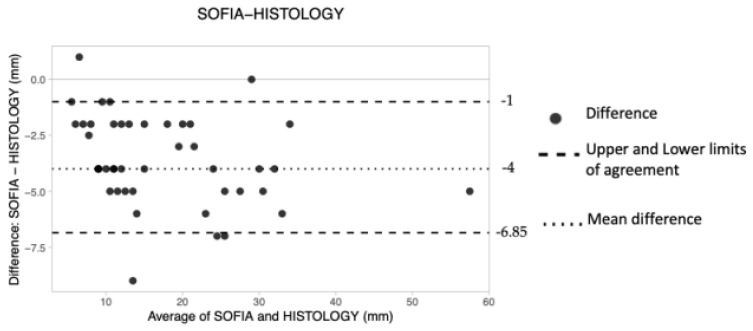
The Bland–Altman scatter plot shows the agreement of measurements between prone 3D ABUS (SOFIA) and histology. Millimeters (mm).

Forty-four preoperative measurements were obtained using prone 3D ABUS (Table 5). The mean difference, upper LoA and lower LoA were −4.00 mm, −1.00 and −6.85, respectively (Table 4), suggesting that the technique tended to consistently underestimate tumor size by approximately 4 mm. A larger measuring error is also shown on the B&A plot, as depicted by the wider space occupied by the dots along the *Y* axis (Figure 4).

##### Magnetic Resonance Imaging (MRI)

The overall *d* was 1 mm, the upper LoA was 5 and the lower LoA was −4.

**Figure 5:**
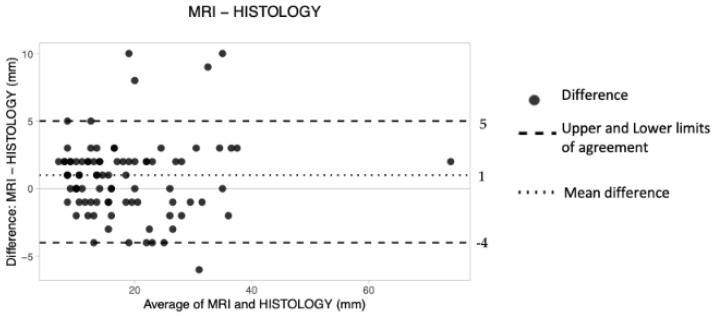
The Bland–Altman scatter plot shows the agreement of measurements between magnetic resonance imaging (MRI) and histology. Millimeters (mm).

These results suggest that preoperative MRI tended to obtain greater measurements of cancer size compared to HE (Table 4). The obtained plot shows an overall good level of agreement, with all data lying within the constructed limits of agreement. However, the dots occupy a wider space along the *Y* axis (Figure 5).

##### Hand-Held Ultrasound (HHUS)

**Figure 6:**
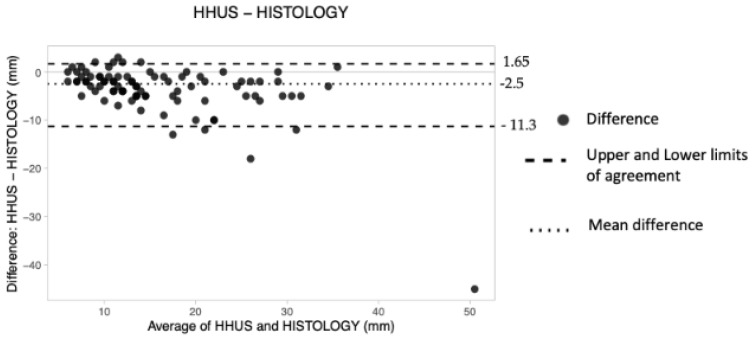
The Bland–Altman scatter plot shows the agreement of measurements between hand-held ultrasound (HHUS) and histology. Millimeters (mm).

The overall mean difference was −2.5 mm, the lower LoA was −11.3 and the upper LoA was 1.65 (Table 4). Therefore, it was concluded that HHUS underestimated tumor size by about 2.5 mm. Finally, no pattern of dot distribution seems to be present in the graph (Figure 6), with approximately six dots lying outside the limits of agreement. The criterion according to which 95% of the data should lie within the LoAs was thus adhered to.

## 4. Discussion

Tumor extension assessment is one of the essential features to consider in the therapeutic approach, especially in the decision of BCS, which determines more patient compliance, and a better prognosis and quality of life after surgery. The standardization of the ABUS procedure, the independence of the operator and the multiplanar reconstruction allow for optimization of the lesion measurement [28].

In this two-center prospective study, we evaluated the agreement of two different types of ABUS in the supine and prone positions, HHUS and CE-MRI with HE (reference standard) in measuring breast cancer size.

Our study revealed a good agreement of all the investigated techniques with the reference standard (95% of data lay within the LoAs). The line of equality was in the interval for all the imaging methods investigated, showing no significant systematic differences.

Supine 3D ABUS exhibited good agreement with HE, with a tendency to underestimate breast cancer size (*d* = −2 mm). It was slightly lower compared to HHUS, with a mean difference (*d*) of −2 m. Our results are in line with the literature, both for the well-known underestimation of US [29] and for the better performance of 3D US compared to HHUS [25].

Prone 3D ABUS revealed the worst agreement with histology, with the mean difference being furthest away from the line of equality (*d* = −4 mm). It tended to underestimate the cancer size more than supine 3D ABUS (*d* = −2 mm), potentially because the images were acquired by the movement of the probe in a clockwise direction in one circular motion, as opposed to supine 3D ABUS which obtains at least three scan views. Unexpectedly, the underestimation of prone 3D US was higher compared to HHUS (*d* = −2.5 mm). This result should be confirmed in future studies with a larger population.

To our knowledge, our study is the first to compare the performance of two different ABUS techniques in evaluating breast cancer tumor size.

Few previous studies have evaluated the agreement of ABUS, HHUS and MRI with histological examination in defining breast cancer size. Girometti et al. [24] confirmed the superiority of MRI compared to supine 3D ABUS, with comparable results in terms of limits of agreement with histology. He added that 3D ABUS was slightly more accurate than CE-MRI (in the subgroup of lesions ≤2 cm), with a better agreement with histology than HHUS (especially for intraductal tumors and lesions >2 cm). Due to the small sample size, we did perform a stratification based on cancer size.

Our results confirm the well-known superiority of MRI (*d* = 1 mm) and the tendency of the technique to overestimate the cancer size [30]. Both the 3D ABUS techniques showed a lower agreement than MRI, in line with the literature.

This prospective study has some limitations. Firstly, the sample differed between the two groups, which may cause confounding variables and, consequently, bias. To avoid and reduce this, we tried to homogenize the sample by enrolling patients without statistically significant differences (*p* > 0.05, Student’s *t*-test), especially regarding the histological tumor size (breast cancers included in the study had a diameter between <10 mm and 30 mm). As previously specified, we used the same modality to measure breast cancer diameter and the same type of reading sessions. Secondly, we did not consider cancer stratification based on molecular subtypes, and the evaluation of the presence of multicentric and multifocal lesions, due to the absence of statistically significant differences, possibly due to the small sample size. Finally, we did not evaluate intra- and interobserver agreement in both groups.

Further studies performed on the same sample, enrolling a more significant number of patients, will help validate our preliminary results.

## 5. Conclusions

Both ABUS techniques appear to be useful tools in breast cancer staging. Supine 3D ABUS is more accurate than prone in the preoperative evaluation of breast cancer size, with a slight tendency toward underestimation, and it could replace the HHUS in breast cancer staging.

## Figures and Tables

**Table 1:** Study population characteristics. Invasive ductal carcinoma (IDC); invasive lobular carcinoma (ILC); no special type (NST); ductal carcinoma in situ (DCIS).

Group	Group 1	Group 2	*p*-Value (Student’s *t*-Test)
**Nb patients**	44	44	-
**Mean age (years)**	56.2 (SD 10.9)	60.5 (SD 10.7)	0.06659
**Type of surgery**			
BCS	40	37	-
Mastectomy	4	7	-
**Histological type**			
IDC	33	36	-
ILC	10	2	-
NST	0	6	-
DCIS	1	0	-
**Molecular subtype**			
Luminal A-like	10	10	-
Luminal B-like HER2+	11	4	-
Luminal B-like HER2−	18	24	-
HER2-positive	2	3	-
Triple-negative	3	3	-
**Mean size at the histology of the surgical specimen (mm)**	16.4 ± SD 7.7 (7–37)	19.5 ± SD 11.9 (6–73)	0.124347

**Table 2:** Mean lesion size as measured by hand-held ultrasound (HHUS), 3D ABUS, contrast-enhanced magnetic resonance imaging (CE-MRI) and histological examination (HE) of the surgical specimen in each group.

Group	*n*	HHUS (mm)	3D ABUS (mm)	CE-MRI (mm)	HE (mm)
Group 1	44	12.4 ± SD 4.9 (5–25)	14.1 ± SD 5.8 (5–30)	16.5 ± SD 6.9 (9–35)	16.4 ± SD 7.7 (7–37)
Group 2	44	16.3 ± SD 8.5 (5–36)	15.6 ± SD 10.1 (5–55)	21.2 ± SD 12.4 (8–75)	19.5 ± SD 11.9 (6–73)

**Table 3:** Individual measurements obtained by supine 3D ABUS (ABVS) and histology (HE), and the agreement between the two methods. Millimeters (mm); mean differences (*d*); standard deviation (SD).

Patient	ABVS (mm)	HE (mm)	Mean	Difference
1	9	7	8	2
2	10	10	10	0
3	11	8	9.5	3
4	9	14	11.5	−5
5	25	27	26	−2
6	11	11	11	0
7	30	37	33.5	−7
8	20	27	23.5	−7
9	13	16	14.5	−3
10	25	35	30	−10
11	19	24	21.5	−5
12	15	15	15	0
13	25	27	26	−2
14	5	10	7.5	−5
15	11	13	12	−2
16	20	27	23.5	−7
17	8	10	9	−2
18	12	10	11	2
19	19	18	18.5	1
20	15	25	20	−10
21	10	13	11.5	−3
22	15	16	15.5	−1
23	20	16	18	4
24	10	24	17	−14
25	9	8	8.5	1
26	15	14	14.5	1
27	9	15	12	−6
28	10	12	11	−2
29	25	30	27.5	−5
30	14	17	15.5	−3
31	18	19	18.5	−1
32	15	16	15.5	−1
33	8	9	8.5	−1
34	9	7	8	2
35	9	10	9.5	−1
36	8	8	8	0
37	10	13	11.5	−3
38	8	8	8	0
39	16	21	18.5	−5
40	11	10	10.5	1
41	18	20	19	−2
42	14	15	14.5	−1
43	18	20	19	−2
44	10	11	10.5	−1
**Mean (*d*)**				−2
**SD**				3

**Table 4:** Difference (*d*) and upper and lower limits of agreement (LoAs) of the comparison between preoperative imaging techniques vs. histological examination (HE) calculated with Bland–Altman analysis. Magnetic resonance imaging (MRI); hand-held ultrasound (HHUS); millimeters (mm).

Supine 3D US (ABVS) vs. HE	Value (mm)	95% CI Lower Limit	95% CI Upper Limit
Difference (*d*)	−2	−3	−1
Upper LoA	2	1	3.85
Lower LoA	−9.55	−13.4	−5.85
**Prone 3D US (SOFIA) vs. HE**			
Difference (*d*)	−4.00	−4.00	−2.5
Upper LoA	−1.00	−2.00	0.85
Lower LoA	−6.85	−8.70	−5.00
**MRI vs. HE**			
Difference (*d*)	1	0	2
Upper LoA	5	3	9.65
Lower LoA	−4	−4	−2
**HHUS vs. HE**			
Difference (*d*)	−2.5	−4	−2
Upper LoA	1.65	0	2
Lower LoA	−11.3	−16.25	−7.65

**Table 5:** Individual measurements obtained by prone 3D ABUS (SOFIA) and histology (HE), and the agreement between the two methods. Millimeters (mm); mean differences (*d*); standard deviation (SD).

Patient	SOFIA (mm)	HE (mm)	Mean	Difference
1	30	36	33	−6
2	10	11	10.5	−1
3	7	11	9	−4
4	12	14	13	−2
5	7	9	8	−2
6	7	6	6.5	1
7	10	12	11	−2
8	9	14	11.5	−5
9	14	16	15	−2
10	10	15	12.5	−5
11	13	17	15	−4
12	11	17	14	−6
13	29	29	29	0
14	5	7	6	−2
15	28	32	30	−4
16	23	28	25.5	−5
17	11	16	13.5	−5
18	28	33	30.5	−5
19	6	8	7	−2
20	17	19	18	−2
21	10	14	12	−4
22	25	30	27.5	−5
23	20	23	21.5	−3
24	30	34	32	−4
25	20	22	21	−2
26	8	13	10.5	−5
27	8	12	10	−4
28	9	10	9.5	−1
29	19	21	20	−2
30	9	13	11	−4
31	7	11	9	−4
32	33	35	34	−2
33	9	13	11	−4
34	5	6	5.5	−1
35	22	29	25.5	−7
36	20	26	23	−6
37	55	60	57.5	−5
38	22	26	24	−4
39	21	28	24.5	−7
40	7	11	9	−4
41	18	21	19.5	−3
42	9	18	13.5	−9
43	6,5	9	7.75	−2.5
44	11	21	16	−10
**Mean (*d*)**				−4
**SD**				2

## Data Availability

Not applicable.
